# Developmental assessments during the first 5 years of life in infants fed breast milk, cow's milk formula, or soy formula

**DOI:** 10.1002/fsn3.1630

**Published:** 2020-05-13

**Authors:** Jayne Bellando, Ginger McCorkle, Beverly Spray, Clark R. Sims, Thomas M. Badger, Patrick H Casey, Holly Scott, Sarah R. Beall, Seth T. Sorensen, Aline Andres

**Affiliations:** ^1^ Arkansas Children's Nutrition Center Little Rock AR USA; ^2^ Department of Pediatrics University of Arkansas for Medical Sciences Little Rock AR USA; ^3^ Arkansas Children's Research Institute Little Rock AR USA; ^4^ Chenal Family Therapy Little Rock AR USA

**Keywords:** childhood, cognition, development, feeding, language, neurodevelopment

## Abstract

**Objective:**

To investigate the effects of infant feeding mode on childhood cognition and language as the differential effects of infant feeding on development remain understudied.

**Methods:**

Breastfed [BF, 174], cow's milk‐based formula‐fed [MF, 169], or soy protein‐based formula‐fed [SF, 161] children were longitudinally tested from age 3 to 60 months for neurodevelopment. Data were analyzed using mixed models while adjusting for multiple covariates. Sex differences were also assessed.

**Results:**

Standard scores were within established norms for all groups. There were no differences in mental development to age 24 months, yet BF children had significantly higher motor development scores at age 3 months than SF children (99.1 versus. 97.2). BF children had significantly higher composite intelligence scores at 48 months than MF and SF children (113.4 versus. 109.6 and 108.4, respectively) and higher verbal intelligence scores than SF children at 48 (105.6 versus. 100.7) and 60 months (109.8 versus. 105.9). Greater total language scores at ages 36 and 48 months were found in BF children compared with children fed MF or SF (*p* < .001), with differences between sexes for auditory comprehension. Higher total language scores at age 60 months were found between BF and SF (105.0 versus. 100.1).

**Conclusion:**

Breastfeeding was associated with small, statistically significant, differences between children ages 3 and 5 years in verbal intelligence, expressive communication, and auditory comprehension with the latter having potential sexual dimorphic effects. Yet, these differences remain small and may not be of clinical relevance. Overall, MF and SF did not significantly differ.

## Graphical abstract

Although breast milk is recommended as the ideal source of nutrition for the first year of life, there is little consensus in the literature regarding long‐term benefits of breastfeeding over cow's milk‐based or soy protein‐based formula feeding to children’s neurodevelopment. We found that while a statistically significant advantage for breastfed children on language and cognitive tests exists, this is small in magnitude and not necessarily clinically relevant. All diets investigated provide adequate nutrients to sustain development up to age 60 months.

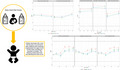

## 1 INTRODUCTION

The American Academy of Pediatrics (AAP) recommends the use of human milk as an ideal source of nutrition for the first year of life and endorses exclusive breastfeeding (BF) until six months of age (American Academy of Pediatrics, 2012; World Health Organization [WHO], 2013). The benefits of BF in decreasing the incidence and severity of various illnesses and sudden infant death syndrome are well documented (American Academy of Pediatrics, 2012). Suggested alternatives to BF, as noted by the AAP, include the use of cow's milk‐based formulas (MF) as a first choice and soy protein‐based formulas (SF) as a second choice (American Academy of Pediatrics, 2012). While there is clear support for the physical health benefits of BF, there is less consensus in the literature on the long‐term relative benefits of BF on language, developmental, and behavioral outcomes. Many previous studies examining the link between breastfeeding and cognition have methodological flaws, such as failing to control for confounders and, therefore, susceptibility bias (Jain, Concato, & Leventhal, 2002).

When accounting for socio‐economic status (SES) and maternal education, a meta‐analysis revealed the difference in points in cognitive function between BF and formula‐fed (FF) children was halved (Anderson, Johnstone, & Remley, 1999). Similarly, a more recent meta‐analysis showed a smaller difference in cognitive scores between BF and FF children age 1–15 years when controlling for maternal intelligence quotient (IQ) (Horta, Loret de Mola, & Victora, 2015). Another review revealed that BF does have a small, but long‐term impact, with higher IQ scores in 5– 6‐year‐old children and lack of cognitive decline in participants aged ~68 years (Horta, de Sousa, & Loret de Mola, 2018). There is less evidence on the impact of BF on language development. Smith, (2015) highlighted that when maternal IQ or vocabulary skills were considered, modest, but significant, differences in language development remained in studies with larger sample sizes, for infants who were fed breast milk (BM) for longer durations, or for infants who were exclusively fed BM (Smith, 2015). There are currently no studies investigating the effect of soy formula (SF) on neurodevelopment in school‐age children. Previous published work exploring differences in outcomes associated with early infant diets that include SF originated from our work on this *Beginnings Study*. The connection between soy and children, found infrequently in research, typically involves the study of low birth weight and preterm infants. Clearly, more research is needed to fully elucidate the impact of early‐life diet on language skills and cognition, specifically including SF in the investigation of developmental outcomes.

The *Beginnings Study*, reported here, investigated whether the growth and development of SF fed infants was comparable to infants fed BM or MF (Andres, Case y, Cleves, & Badger, 2013; Andres et al., 2012, 2015; Gilchrist, Moore, Andres, Estroff, & Badger, 2010; Pivik et al., 2015; Pivik, Dykman, Jing, Gilchrist, & Badger, 2007). Particularly, this report examined differences in the first 5 years of life of children who were fed BM, MF, or SF as infants with respect to sex, race, gestational age, maternal and paternal education, maternal IQ, and cohesion score, which have been identified as important variables to consider when examining cognitive and language skills. It was hypothesized that there would be differences in cognitive and language skills between feeding groups, with BF children having statistically higher scores in cognitive and language performance at one or more ages tested. No significant differences on the assessments administered were expected at any age between the MF and SF infants.

## 2 MATERIALS AND METHODS

### 2.1 Participants

Participants were 504 eligible infants enrolled between 2002 and 2011 (www.clinicaltrials.gov, ID # NCT00616395) and were followed until age 6 years (the last study visit was July, 2017). Participants were recruited when infants were between ages 1 and 2 months. Pregnancies were uncomplicated with no medical diagnoses (e.g., diabetes and preeclampsia) or medications known to affect fetal or infant growth and development (e.g., selective serotonin reuptake inhibitors and thyroid replacement). All mothers were nonsmokers, denied alcohol use during pregnancy and reported no use of soy products or other potential estrogenic compounds during pregnancy and/or lactation. Infants were term (>37 weeks), between 2.7 kg (6 lbs) and 4.1 kg (9 lbs) at birth, had no known medical diagnoses and had not been administered medications known to affect growth or development. Other exclusion criteria included: change of formula after age 2 months and before age 12 months; complementary foods before age 4 months (to comply with the American Academy of Pediatrics (AAP) guidelines at the time of the study enrollment); and body weight at age 3 months less than 5 kg (11 lbs). Study visits included in this report were conducted at 3, 12, 24, 36, 48, and 60 months. Consent was obtained prior to study procedures. The study was approved by the institutional review board.

### 2.2 Diets

Parents made decisions about which diet to feed their infants prior to enrollment. In this study, 174 children were BF, 169 were fed MF, and 161 were fed SF. Enrollment was performed in a diet‐type paced manner (i.e., for every BF infant enrolled, one MF and one SF were enrolled) to ensure equal distribution across feeding groups throughout the study period. Those electing to formula feed chose between milk‐based formulas (Similac^®^ Advance^®^ or Enfamil^®^ Lipil^®^) or soy protein‐based formulas (Similac^®^ Soy Isomil^®^ or Enfamil^®^ Prosobee^®^) formulas. Similac formulas were manufactured by Abbott Nutrition, Columbus, OH and Enfamil formulas were manufactured by Mead Johnson, Evansville, IN. All formulas contained supplemental docosahexaenoic acid (DHA) and arachidonic acid (ARA) and were provided to the participants. All formula‐fed (FF) infants remained on their selected formula until 12 months of age. For breastfeeding (BF) infants, breastfeeding was encouraged until age 12 months. If not possible, BF infants were weaned to cow's milk‐based formula (MF) between 6 and 12 months. Among the BF infants, 56% were breastfed until age 12 months, 23% switched to MF between 9 and 12 months, and 21% switched to MF before 9 months of age. None of the BF infants were switched to SF. Complementary foods (e.g., juices, cereals and solid foods) could be introduced after age 4 months for all diet groups.

### 2.3 Demographics

Family demographics were obtained at enrollment. Information about child's sex, race, marital status, parental education, mother's age, and parental income was obtained on self‐administered questionnaires. Mothers reported information about child's birth weight, birth length, gestational age, and developmental or mental health disorders.

### 2.4 Study visits

Licensed psychological examiners, supervised by a licensed psychologist and blinded to the study groups, administered all measures. Examiners held a minimum of a Master's Degree, had specialized training in psychometric testing and were licensed by the Arkansas Board of Psychology.

#### 2.4.1 Wechsler abbreviated scale of intelligence (WASI)

The WASI, an abbreviated scale of intellectual performance that measures verbal (Verbal Intelligence Quotient index), visual (Performance Intelligence Quotient index), and general cognitive abilities (Full Scale Intelligence Quotient index [FSIQ]), was administered to mothers at the 3‐month study visit (Wechsler, 1999).

#### 2.4.2 Symptom assessment‐45 questionnaire (SA‐45)

The SA‐45, a brief general assessment of psychiatric symptomatology that yields a Global Severity Index (GSI), was administered to mothers at the 3‐month study visit (Davison et al., 1997).

#### 2.4.3 Family adaptability and cohesion evaluation scale‐II (FACES‐II)

The FACES‐II, a self‐report scale of perceptions of cohesion and adaptability in the family system across the life cycle, was administered to mothers at the 3‐month study visit. Cohesion, defined as the emotional bonding that family members have toward one another, and Adaptability, defined as the ability of a marital or family system to change its power structure in response to stress, are reported (Olson, Portner, & Bell, 1982).

#### 2.4.4 Bayley scales of infant development ‐ 2nd edition (BSID‐2)

The BSID‐2 was administered to assess the Mental Development Index (MDI) and Psychomotor Development Index (PDI) at 3, 12, and 24 months of age (Bayley, 1993).

#### 2.4.5 The preschool language scale‐3 (PLS‐3)

The PLS‐3 was administered to children at each study visit. Total Language Score (TLS), Auditory Comprehension (AC), and Expressive Communication (EC) results are reported (Zimmerman, Steiner, & Pond, 1992).

#### 2.4.6 The Reynolds intellectual assessment scales (RIAS)

The RIAS was administered at the 48‐ and 60‐month study visits to measure general cognitive ability [Composite Intelligence Index (CIX)] as well as verbal [Verbal Index (VIX)] and nonverbal intelligence [Nonverbal Index (NIX)] (Reynolds & Kamphaus, 2003).

### 2.5 Statistical analysis

Participant characteristics measured on a continuous scale were summarized as means and standard deviations and compared across feeding groups with a one‐way ANOVA. Participant characteristics measured categorically were summarized as counts and percentages and compared across feeding groups using Pearson's *x* ^2^ test or Fisher's exact test. In addition, results were examined for significant effects of sex. Longitudinal levels of psychological and language measures (standard scores as reported elsewhere (Andres et al., 2012) by feeding group were assessed by mixed models (REML method) with unstructured covariance (yielding lowest BIC), adjusting for sex, race, gestational age, maternal and paternal education, maternal IQ, and cohesion score as main effects. Covariates were selected based upon prior reported effects and significance in the model. Marital status was evaluated and redundant (i.e., shared variance) with marital education and because marital education had a stronger relationship to the outcome variables it was kept in the model and marital status was removed. Slice tests at each study visit were performed and p‐values for feeding group comparisons were model‐wise Sidak‐adjusted to control the type I error rate. Corresponding least squares means and their standard errors at study visits were obtained. Statistical tests were considered statistically significant at a prespecified 0.05 level. Analyses were performed in SAS software, version 9.4 (SAS Institute).

## 3 RESULTS

**Figure 1:**
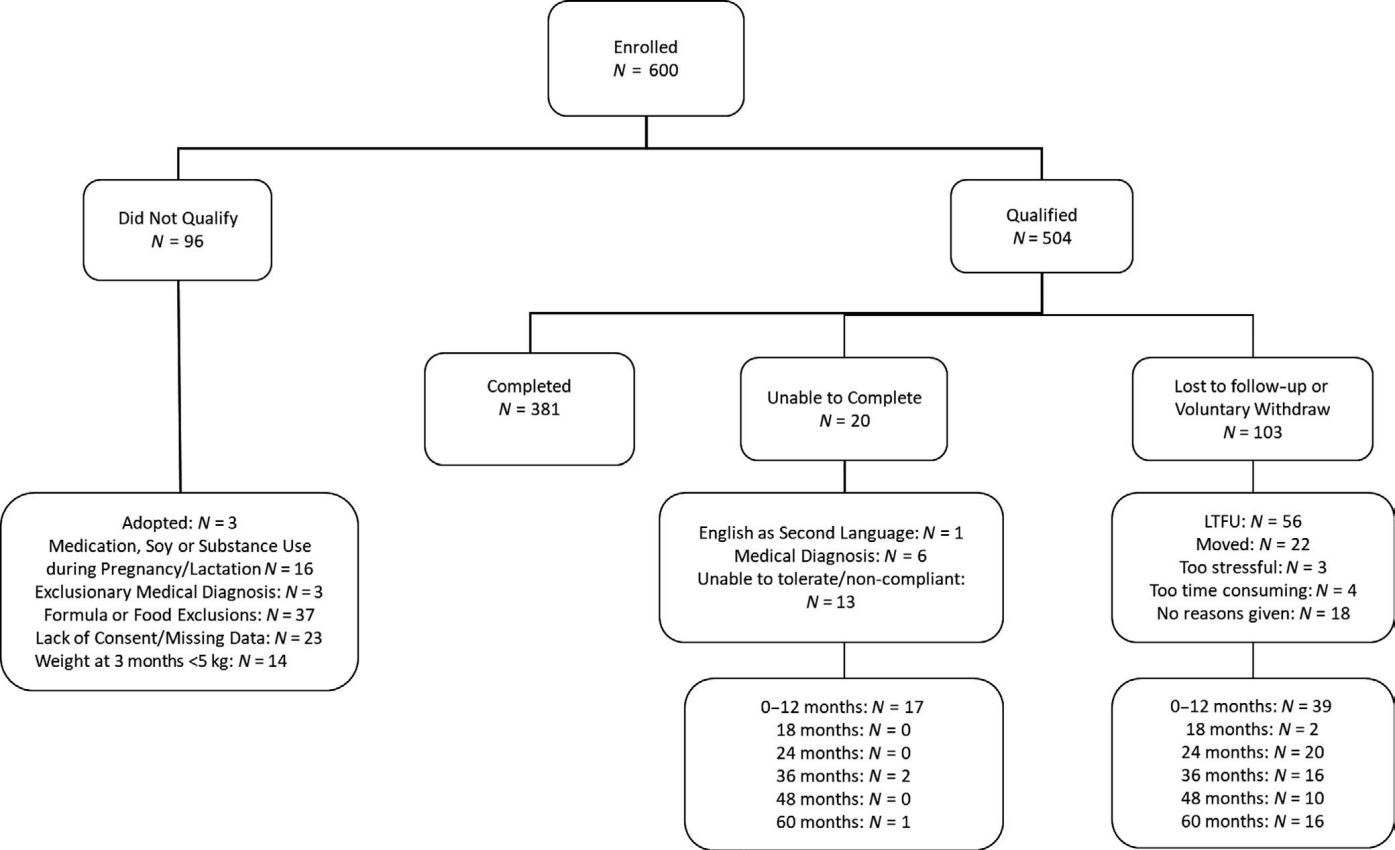
Flowchart of the study population

A total of 600 infant‐mother pairs were enrolled between September 2002 and September 2011 (Figure 1). Of these, 96 (16%) did not qualify and 103 (17%) were either lost to follow‐up or voluntarily withdrew. Of the participants who voluntarily withdrew, 47% moved from the Central Arkansas area during the study period. The remaining 381 (64%) children completed the study through age 5 years. Assessment results are reported as standard scores having a mean or average score of 100 and a standard deviation of 15. Therefore, average performance is characterized with scores that fall within the range of 85 – 115.

### 3.1 Demographic and maternal measures

About 88% of enrolled mothers were Caucasian, most (93%) were married or cohabitating and 74% graduated from high school or had some college or technical school training (Table 1). Fathers of BF infants had higher incidence of graduate school education (18%) compared with fathers of MF or SF fed infants (13 and 7%, respectively). There were no significant differences between groups in child sex, household income, or age of mother at delivery. For the total sample, mothers’ mean cognitive scores [Full Scale Intelligence Quotient (FSIQ)] were in the average range (105.9 ± 10.6 (Wechsler, 1999), although there was a significant difference with mothers of BF infants scoring higher than mothers of formula‐fed (FF) infants. BF infants had a modest, but significantly, longer gestational period compared with MF and SF infants (by 2.8 and 3.5 days, respectively, Table 1). The number of children diagnosed with developmental or mental health disorders during the study was not significantly different between feeding groups (see Table, Supplemental Digital Content 1).

**Table 1:** Participant characteristics

	All	Breast	Milk	Soy	*p‐value*
*N*	504	174	169	161	
**Race, *N* (%)**					**.002**
African American	46 (9.1)	7 (4.0)	14 (8.3)	25 (15.5)	
Caucasian	442 (87.7)	165 (94.8)	147 (87.0)	130 (80.8)	
Other	6 (3.2)	2 (1.2)	8 (4.7)	6 (3.7)	
**Sex, *N* (%)**					.689
Girls	238 (47.2)	86 (49.4)	80 (47.3)	72 (44.7)	
Boys	266 (52.8)	88 (50.6)	89 (52.7)	89 (55.3)	
**Gestational age, weeks (*SD*)**	39.3 (1.0)	39.6 (1.0)	39.2 (0.9)	39.1 (1.0)	**.001**
**Maternal age at delivery, years (*SD*)**	29.6 (4.6)	29.2 (4.2)	29.8 (4.8)	29.7 (4.8)	0.511
**Maternal FSIQ, score (*SD*)**	105.9 (10.6)	109.5 (10.2)	105.1 ( 9.2)	102.8 (11.4)	**<.001**
**Maternal education, *N* (%)**					**<.001**
Less than high school degree	2 (0.4)	1 (0.6)	1 (0.6)	0 (0)	
High school/ GED	180 (35.7)	41 (23.6)	59 (34.9)	80 (49.7)	
College degree	192 (38.1)	77 (44.2)	74 (43.8)	41 (25.5)	
Graduate degree/ training	124 (24.6)	51 (29.3)	33 (19.5)	40 (24.8)	
Missing	6 (1.2)	4 (2.3)	2 (1.2)	0 (0)	
**Paternal education, *N* (%)**					**<.001**
Less than high school degree	6 (1.2)	2 (1.2)	2 (1.2)	2 (1.2)	
High school/ GED	215 (42.7)	49 (28.1)	79 (46.7)	87 (54.1)	
College degree	187 (37.1)	83 (47.7)	53 (31.4)	51 (31.7)	
Graduate degree/ training	64 (12.7)	31 (17.8)	22 (13.0)	11 (6.8)	
Missing	32 (6.3)	9 (5.2)	13 (7.7)	10 (6.2)	
**Marital status, *N* (%)**					**.041**
Married/Cohabitating	470 (93.2)	165 (94.8)	155 (91.7)	150 (93.2)	
Single/Separated/Never married	26 (5.2)	4 (2.3)	11 (6.5)	11 (6.8)	
Missing	8 (1.6)	5 (2.9)	3 (1.8)	0 (0.0)	
**Maternal household income, *N* (%)**					**<.001**
Less than $20,000	97 (19.3)	31 (17.8)	28 (16.6)	38 (23.6)	
$20,000‐$59,999	203 (40.3)	48 (27.6)	86 (50.9)	69 (42.8)	
Greater or equal to $60,000	23 (4.6)	9 (5.2)	5 (3.0)	9 (5.6)	
Missing	181 (35.9)	86 (49.4)	50 (29.6)	45 (28.0)	
**Paternal household income, *N* (%)**					0.6
Less than $20,000	32 (6.4)	9 (5.2)	8 (4.7)	15 (9.3)	
$20,000‐$59,999	284 (56.4)	102 (58.6)	94 (55.6)	88 (54.7)	
Greater or equal to $60,000	124 (24.6)	44 (25.3)	43 (25.5)	37 (23.0)	
Missing	64 (12.6)	19 (10.9)	24 (14.2)	21 (13.0)	

Note

Data presented as counts (%) or mean (*SD*), FSIQ: full scale intelligence quotient.

Bold *p*‐values represent those findings that are significant.

As shown in Table 2, there was no significant difference between mothers on the measure of psychiatric symptomatology as indicated by the Global Severity Index (GSI) of the Symptom Assessment‐45 Questionnaire (SA‐45). The Cohesion score of the measure of family emotional environment was significantly higher in BF compared with MF and SF infants (Table 2). There was no difference in the Adaptability score. All statistical models were adjusted for variables that were significantly different between groups, including maternal intelligence quotient (IQ), gestational age, and cohesion scores.

**Table 2:** Mother affect (SA‐45) and family environment (FACES‐II)^1^

	Breast	Milk	Soy	*p*‐value
Global Severity Index score	48.0 (6.6)	48.5 (7.9)	48.9 (8.1)	.516
Cohesion score	7.1 (1.0)_a_	6.6 (1.4)_b_	6.5 (1.4)_b_	**.003**
Adaptive Score	5.5 (1.1)	5.4 (1.4)	5.1 (1.3)	.110

^1^ Reported as Mean (*SD*) at the 3 month visit. Means with different subscript letters within a row differ significantly at the 0.05 level.

Bold *p*‐values represent those findings that are significant.

### 3.2 Developmental and cognitive measures

#### 3.2.1 Developmental results

The Mental Development Index (MDI) scores from the Bayley Scales of Infant Development—2nd Edition (BSID‐2) were in the average range and did not differ significantly between feeding groups (Table 3). There were significant differences in the Psychomotor Development Index (PDI) scores between feeding groups at 3 months, with BF infants having higher scores than SF infants. Children fed MF during infancy did not differ from BF or SF children (Table 3). There were no significant effects of sex.

**Table 3:** Least squares means for Bayley scales of infant development—2nd edition mental and motor development index^1^

	Age	Breast		Milk		Soy		*p*‐value
		*N*	Mean (*SEM*)	*N*	Mean (*SEM*)	*N*	Mean (*SEM*)	
Mental development index	3 months	173	101.2 (0.9)	167	101.6 (0.9)	158	101.0 (0.9)	.632
	12 months	158	102.4 (1.0)	150	100.2 (1.0)	148	101.1 (1.0)	.084
	24 months	145	100.5 (1.2)	127	98.9 (1.3)	129	98.6 (1.2)	.316
Psychomotor development Index	3 months	172	99.1 (1.0)_a_	166	98.1 (1.0)_ab_	158	97.2 (1.0)_b_	**.043**
	12 months	157	97.5 (1.3)	150	97.3 (1.3)	148	99.0 (1.3)	.424
	24 months	140	103.8 (1.2)	126	101.9 (1.2)	129	102.2 (1.2)	.204

^1^ Adjusted for sex, race, gestational age, maternal and paternal education, maternal intelligence quotient, and cohesion score. Least squares means (*SEM*) with different subscript letters within a row differ significantly at the *p* < .05 level. P‐values were Sidak‐adjusted to control for model‐wise type I error rates.

Bold *p*‐values represent those findings that are significant.

#### 3.2.2 Intelligence results

There were significant differences in the Composite Intelligence Index (CIX) of the Reynolds Intellectual Assessment Scales (RIAS) between feeding groups at 48 months, with BF children having higher scores versus MF and SF children (3.8 and 5 points, respectively, Table 4). Children fed MF during infancy did not differ from children fed SF. A significant difference was also found between feeding groups in the Verbal Intelligence Index (VIX) at 48 and 60 months, with BF children having higher scores (~4.4 points on average) than children fed SF (Table 4). Children fed MF during infancy did not differ from children fed BF or SF. There were no differences in the Nonverbal Intelligence Index (NIX) between feeding groups at 48 or 60 months.

**Table 4:** Least squares means of the Reynolds intellectual assessment scales composite score at 4 and 5 years of age^1^

	Age	Breast		Milk		Soy		*p*‐value
		*N*	Mean (*SEM*)	*N*	Mean (*SEM*)	*N*	Mean (*SEM*)	
Composite intelligence index	48 months	109	113.4 (2.4)_a_	93	109.6 (2.3)_b_	95	108.4 (2.4)_b_	**.016**
	60 months	134	115.0 (2.3)	107	113.3 (2.3)	102	110.8 (2.4)	.055
Verbal intelligence index	48 months	112	105.6 (2.4)_a_	94	102.2 (2.3)_ab_	96	100.7 (3.3)_b_	**.020**
	60 months	134	109.8 (2.4)_a_	107	106.6 (2.3)_ab_	102	105.9 (3.4)_b_	**.045**
Nonverbal intelligence index	48 months	113	121.2 (2.7)	94	117.1 (2.6)	98	117.5 (2.7)	.099
	60 months	134	118.8 (2.7)	108	118.7 (2.6)	104	115.4 (2.7)	.172

^1^ Adjusted for sex, race, gestational age, maternal and paternal education, maternal intelligence quotient, and cohesion score. Least squares means (*SEM*) with different subscript letters within a row differ significantly at the *p* < .05 level. *p*‐values were Sidak‐adjusted to control for model‐wise type I error rates.

Bold *p*‐values represent those findings that are significant.

### 3.3 Language results

#### 3.3.1 Total language score (TLS)

BF children had significantly higher total scores (~6 points on average) on the Preschool Language Scale‐3 (PLS‐3) than children fed MF or SF at ages 36 and 48 months (Table 5). At age 60 months, BF infants scored significantly higher than SF, but SF and MF children did not differ significantly. There was no significant effect of sex in TLS, and scores did not differ significantly by feeding group among participants at younger ages.

**Table 5:** Preschool language scale‐3 least squares means for total standard score, auditory comprehension and expressive communication^1^

	Age	Breast		Milk		Soy		*p*‐value
		*N*	Mean (*SEM*)	*N*	Mean (*SEM*)	*N*	Mean (*SEM*)	
Total standard score	3 months	163	97.9 (1.1)	147	96.7 (1.0)	149	98.3 (1.1)	.280
	12 months	152	97.2 (1.0)	135	96.9 (1.0)	135	98.5 (0.9)	.144
	24 months	141	97.2 (1.3)	116	94.0 (1.3)	116	95.0 (1.3)	.087
	36 months	128	104.2 (1.4)_a_	106	97.9 (1.4)_b_	97	98.0 (1.5)_b_	**<.001**
	48 months	127	105.8 (1.4)_a_	105	102.1 (1.4)_b_	98	99.4 (1.5)_b_	**<.001**
	60 months	130	105.0 (1.3)_a_	105	102.1 (1.4)_ab_	99	100.1 (1.4)_b_	**.010**
Auditory comprehension	3 months	163	95.4 (1.1)	148	94.9 (1.0)	149	95.1 (1.0)	.884
	12 months	152	95.8 (1.0)	135	95.7 (1.0)	135	97.5 (1.0)	.103
	24 months	143	97.5 (1.3)_a_	117	92.9 (1.3)_b_	118	94.4 (1.3)_b_	**.009**
	36 months	131	106.4 (1.4)_a_	114	100.2 (1.4)_b_	101	100.9 (1.5)_b_	**<.001**
	48 months	132	106.9 (1.4)_a_	111	102.4 (1.4)_b_	101	100.1 (1.5)_b_	**<.001**
	60 months	132	105.7 (1.4)_a_	107	104.1 (1.4)_ab_	102	101.2 (1.5)_b_	**.032**
Expressive communication	3 months	163	100.8 (1.3)	147	99.0 (1.3)	149	102.0 (1.3)	.100
	12 months	152	98.7 (1.1)	136	98.6 (1.1)	135	99.6 (1.0)	.499
	24 months	142	97.0 (1.3)	118	96.1 (1.3)	117	96.8 (1.3)	.787
	36 months	131	101.2 (1.5)_a_	107	95.7 (1.5)_b_	98	96.2 (1.5)_b_	**.002**
	48 months	128	103.5 (1.4)_a_	106	101.2 (1.5)_ab_	99	99.1 (1.5)_b_	**.037**
	60 months	131	103.3 (1.4)_a_	107	99.3 (1.4)_b_	100	99.5 (1.5)_b_	**.018**

^1^ Adjusted for sex, race, gestational age, maternal and paternal education, maternal intelligence quotient, and cohesion score. Least squares means (*SEM*) with different subscript letters within a row differ significantly at the *p* < .05 level. *p*‐values were Sidak‐adjusted to control for model‐wise type I error rates.

Bold *p*‐values represent those findings that are significant.

#### 3.3.2 Auditory comprehension (AC) and expressive communication (EC)

Differences were found in AC scores between feeding groups at later ages, with BF infants having significantly higher scores (mean average of 5.1 points) than FF infants at ages 24, 36, and 48 months (Table 5). MF and SF children did not differ significantly (Table 5).

When AC scores were stratified by sex, significant differences were found for boys at 24 and 36 months with BF boys having higher AC scores than FF boys. BF girls had higher AC scores than SF girls at 36, 48, and 60 months (Table 6). There was no significant effect of sex in EC.

**Table 6:** Least squares means of preschool language scale‐3 auditory comprehension scores stratified by sex^1^

**Male**	Breast		Milk		Soy		*p*‐value
	*N*	Mean (*SEM*)	*N*	Mean (*SEM*)	*N*	Mean (*SEM*)	
3 months	81	96.5 (1.4)	77	95.9 (1.4)	82	94.7 (1.4)	.424
12 months	72	95.5 (1.4)	71	94.8 (1.3)	74	96.6 (1.3)	.337
24 months	67	96.2 (1.8)_a_	58	89.3 (1.8)_b_	65	90.6 (1.7)_b_	**.003**
36 months	58	104.4 (2.0)_a_	55	95.1 (2.0)_b_	49	99.5 (2.1)_b_	**<.001**
48 months	58	104.7 (2.0)	53	99.2 (2.0)	49	100.6 (2.0)	.059
60 months	58	104.2 (2.1)	51	99.5 (2.1)	49	100.8 (2.1)	.171

Female	Breast		Milk		Soy		*p*‐value
	*N*	Mean (*SEM*)	*N*	Mean (*SEM*)	*N*	Mean (*SEM*)	
3 months	82	93.0 (1.6)	71	92.8 (1.5)	67	93.8 (1.6)	.758
12 months	80	94.8 (1.6)	64	95.9 (1.6)	61	96.8 (1.6)	.363
24 months	76	97.5 (1.9)	59	96.0 (2.0)	53	97.5 (2.1)	.735
36 months	73	106.7 (2.0)_a_	59	104.8 (2.0)_ab_	52	101.0 (2.1)_b_	**.045**
48 months	73	107.7 (2.0)_a_	58	105.1 (2.1)_a_	50	98.5 (2.2)_b_	**<.001**
60 months	73	105.2 (1.9)_a_	56	108.0 (2.0)_a_	49	100.5 (2.1)_b_	**.005**

^1^ Adjusted for race, gestational age, maternal and paternal education, maternal intelligence quotient, and cohesion score. Least squares means (*SEM*) with different subscript letters within a row differ significantly at the *p* < .05 level. *p*‐values were Sidak‐adjusted to control for model‐wise type I error rates.

Bold *p*‐values represent those findings that are significant.

To consider the potential effect of breastfeeding prior to formula feeding and the length of breastfeeding, sensitivity analyses were conducted, which included the age of complementary food introduction. Results were unchanged for children who were fed formula from birth without any exposure to breastmilk, except for MDI and PDI at age 24 months. At age 24 months, BF children had higher MDI scores compared with MF (*p* = .001) and SF (*p* = .016) children. Additionally, higher PDI scores were found in BF children compared with those fed SF (*p* = .02). No significant differences were noted between BF and MF or MF and SF. Results were also unchanged for children who were breastfed until 12 months of age.

Unadjusted developmental, intellectual, and language results are presented as well (**Tables** **S1** **–S3**). Findings revealed support the argued importance of adjusting for confounding variables that greatly affect study outcomes. Crude associations are evident due to the greater variation present without covariate modeling. Results can actually be attributed to factors other than feeding group, such as maternal IQ or socio‐economic status. Such results suggest that the benefit of BM over MF and SF is much more significant than it actually is when important confounders are considered and included in the analysis.

## 4 DISCUSSION

We characterized the developmental, language, and intellectual performances of children age 3 to 60 months who were fed breast milk (BM), cow's milk‐based formula (MF), and soy protein‐based formula (SF) during infancy. To our knowledge, this is the first prospective, comprehensive report of family characteristics and children's neurodevelopmental performances comparing postnatal diet groups including soy‐based formulas while considering major confounders.

Results demonstrated that mean scores on all administered tests were within published normal limits regardless of diet, and the number of children diagnosed with developmental or mental health disorders during the study was not significantly different between feeding groups. Thus, all infant diets investigated in this study provided adequate nutrients to sustain development in children between ages 3–60 months.

In this cohort, breastfeeding (BF) infants were more likely to be part of families whose mothers had significantly higher full‐scale intelligence quotients (IQs), whose parents were married or cohabitating, and whose fathers had higher levels of education. Previous publications are in line with results (Der, Batty, & Deary, 2006; Hendricks, Briefel, Novak, & Ziegler, 2006; Singh, Kogan, & Dee, 2007); however, there is scarce literature examining the paternal education of breastfeeding families. BF was also associated with a longer gestational period compared to formula‐fed (FF) children, although differences were small (3.15 days on average).

Families of children fed MF and SF had lower Cohesion scores compared with families of BF infants, suggesting that families of FF children may have lower emotional bonding between members. Yet, these differences were very small, ~0.6 points lower, indicating results should be interpreted cautiously. These results are in line, though, with findings that mothers’ and fathers’ sensitivity, positive regard, and cognitive stimulation were associated with higher cognitive and language scores as measured by the Bayley Scales of Infant Development—2nd Edition (BSID‐II) Mental Development Index (MDI) and Peabody Picture Vocabulary Test (PPVT) in their children at ages 24 and 36 months (Tamis‐LeMonda, Shannon, Cabrera, & Lamb, 2004) It is possible that family cohesion could have some long‐term impact on the cognitive and language development of children.

A significant difference in motor development was found between BF and SF infants at age 3 months, which is consistent with previously published results during the first year of life (Andres et al., 2012) The analyses showed no differences in mental development between feeding groups, which is in contrast with previous results demonstrating advantages of BF on cognitive function from age 6 to 23 months (Anderson et al., 1999; Choi, Kang, & Chung, 2018; Eickmann et al., 2007). Smaller sample sizes or the lower predictive validity for cognitive function of the BSID‐II assessment during infancy may account for the different results (Camp, 2006; Hack et al., 2005; Wainright & Colombo, 2006). Indeed in this cohort, although BSID‐II MDI scores at 12 and 24 months were significantly and positively correlated to the Reynolds Intellectual Assessment Scales (RIAS) Composite Intelligence Index (CIX) scores at age 5 years (*R* = .27, *p* < .0001 and *R* = .45, *p* < .0001; respectively), the correlation coefficient was low, validating the low predictive values referenced by previous studies.

Although 4‐year‐old FF children scored significantly lower on the CIX than children fed BM, this difference was not sustained at age 5 years. The difference seems to be driven by lower scores in verbal intelligence, which requires accessing and applying prior learning to solve language‐related tasks. Our results are similar to previous studies reporting beneficial effects of BF on intelligence (Horta et al., 2015), but these studies generally focused on differences between breastfeeding and cow's milk formula feeding or the duration of exclusive breastfeeding and not on soy formulas. Although differences were often identified as “small in magnitude,” they did persist into adulthood (Horta et al., 2018; Horwood & Fergusson, 1998; Mortensen, Michaelsen, Sanders, & Reinisch, 2002; Wigg et al., 1998). Further investigation will be needed to determine if a difference in intellectual ability is observed in later life in this specific cohort.

In the presented cohort, the Total Language Score (TLS) of the Preschool Language Scale‐3 (PLS‐3) was significantly lower in children fed MF or SF compared with children fed BM. Although the clinical significance of such findings is yet to be established due to the small differences observed (~6 points), these results suggest a slight advantage for BF children in language development. Analyses of the subscales revealed differences in Auditory Comprehension (AC) and Expressive Communication (EC). There are few studies in the literature that measured language outcomes in relation to feeding practices. Cai et al., (2015) found a positive association between language domain scores on the Bayley Scales of Infant Development—3rd Edition (BSID‐III) and breastfeeding (Cai et al., 2015) Given the results of this study showing differences in expressive and receptive language scores between feeding groups, further investigation of language development according to early feeding practices is warranted. Specifically, future testing of this cohort to examine whether BF children continue to display higher language abilities than their FF peers in later childhood or adolescence would be of clinical interest.

A significant effect of sex was found with BF boys having higher AC scores than FF boys, and BF girls having higher AC scores than SF girls. However, overall findings of this study reveal negligible sex effects on other developmental, total language, and cognitive skills. A systematic review conducted by Etchell et al. (2018) found that the magnitude of sexual dimorphism in language development is not as considerable as once thought and depends on the developmental stages studied (Etchell et al., 2018). Ardila et al. (2011) demonstrated that gender differences during cognitive development are marginal, present in very few test tasks, and responsible for meager variance in scores in children ages 5 to 16 (Ardila, Rosselli, Matute, & Inozemtseva, 2011). In summary, this investigation and others suggest that sexual dimorphism in child cognitive and language development and skill acquisition is not a substantial concern.

This study is strengthened by the large sample size of children from 3 months to 5 years old. Unique to this study is the investigation of three feeding groups to include not only those choosing breastmilk or cow's milk‐based formula, but those opting for soy‐based formula as well. The analysis is enhanced by the inclusion of important covariates and consideration of family structure and maternal factors. The longitudinal assessment of the language skills of these children is a strong asset, as is the longitudinal research design of the project overall. These results are, however, limited by the observational characteristics of the study, which reflect the infant feeding practices of our community. Given that participants were part of one of three feeding groups as predetermined by their parents, the study was not a randomized controlled trial, which should be considered a limitation. The high representation of Caucasian and well‐educated individuals may also limit the generalizability of our findings.

In summary, all feeding groups scored within normal limits for all assessments, indicating that formula feeding does not result in clinically detrimental outcomes with respect to cognitive function and language development. While results suggested a statistically significant advantage for BF children on tests of language and cognition with differences in performance noted between feeding groups to age 5 years, these are very small in magnitude and not of clinical relevance at these ages. SF infants had lower motor development at age 3 months and lower language development at age 5 years compared to BF children, with MF children not differing from either groups on these measures. The consequences and potential mechanisms underlying these small, but statistically significant, differences remain to be established in follow‐up studies of this cohort at later ages.

## CONFLICT OF INTEREST

The authors declare that they do not have any conflict of interest.

## ETHICAL APPROVAL

This study was approved by the Institutional Review Board of the University of Arkansas for Medical Sciences.

## INFORMED CONSENT

Written informed consent was obtained from all study participants.

## Supporting information

Table S1‐S3

Table, Supplemental Digital Content 1
