# Supplementary material for: Developmental assessments during the first 5 years of life in infants fed breast milk, cow's milk formula, or soy formula
Source: Food Sci Nutr. 2020 May 13;8(7):3469–78. doi: 10.1002/fsn3.1630 (PMC7382202; doi:10.1002/fsn3.1630)
Supplement: Supplementary file 1 — Table S1‐S3 [file FSN3-8-3469-s001.docx]

**Supplementary Table 1: Unadjusted Least Squares Means for** **Bayley Scales of Infant Development - 2nd Edition Mental and Motor Development Index**

|  | Age | Breast | | Milk | | Soy | | p-value |
| --- | --- | --- | --- | --- | --- | --- | --- | --- |
|  |  | N | Mean (SEM) | N | Mean (SEM) | N | Mean (SEM) |  |
| MENTAL DEVELOPMENT INDEX | 3 months | 173 | 101.9 (0.4) | 167 | 102.0 (0.5) | 158 | 101.2 (0.5) | 0.368 |
|  | 12 months | 158 | 103.2 (0.7) _a_ | 150 | 100.6 (0.7)_b_ | 148 | 101.5 (0.7)_b_ | **0.025** |
|  | 24 months | 145 | 101.3 (1.0) | 127 | 99.3 (1.1) | 129 | 98.6 (1.1) | 0.131 |
| PSYCHOMOTOR DEVELOPMENT INDEX | 3 months | 172 | 98.1 (0.5)_a_ | 166 | 97.1 (0.5)_b_ | 158 | 96.3 (0.5)_b_ | **0.032** |
|  | 12 months | 157 | 96.6 (1.0) | 150 | 96.3 (1.0) | 148 | 98.3 (1.0) | 0.347 |
|  | 24 months | 140 | 102.9 (0.8) | 126 | 100.8 (0.8) | 129 | 101.3 (0.8) | 0.138 |

Least squares means (SEM) with different subscript letters within a row differ significantly at the p<0.05 level. P-values were Sidak-adjusted to control for model-wise type I error rates.

**Supplementary Table 2: Unadjusted Least Squares Means of The Reynolds Intellectual Assessment Scales Composite Score at 4 and 5 years of age**

|  | Age | Breast | | Milk | | Soy | | p-value |
| --- | --- | --- | --- | --- | --- | --- | --- | --- |
|  |  | N | Mean (SEM) | N | Mean (SEM) | N | Mean (SEM) |  |
| Composite Intelligence Index | 48 months | 109 | 113.9 (1.3)_a_ | 93 | 108.5 (1.3)_b_ | 95 | 106.8 (1.3)_b_ | **< 0.001** |
|  | 60 months | 134 | 115.3 (1.2)_a_ | 107 | 111.7 (1.2)_b_ | 102 | 109.4 (1.3)_b_ | **0.003** |
| Verbal  Intelligence Index | 48 months | 112 | 107.0 (1.2)_a_ | 94 | 102.0 (1.3)_b_ | 96 | 100.0 (1.3)_b_ | **< 0.001** |
|  | 60 months | 134 | 111.0 (1.2)_a_ | 107 | 106.2 (1.2)_b_ | 102 | 105.1 (1.3)_b_ | **0.001** |
| Nonverbal Intelligence Index | 48 months | 113 | 120.1 (1.4)_a_ | 94 | 114.9 (1.4)_b_ | 98 | 114.9 (1.5)_b_ | **0.012** |
|  | 60 months | 134 | 117.7 (1.3) | 108 | 115.5 (1.4) | 104 | 113.2 (1.5) | 0.080 |

Least squares means (SEM) with different subscript letters within a row differ significantly at the p<0.05 level. P-values were Sidak-adjusted to control for model-wise type I error rates.

**Supplementary Table 3: Unadjusted Preschool Language Scale-3 Least Squares Means for Total Standard Score, Auditory Comprehension and Expressive Communication^1^**

|  | Age | Breast | | Milk | | Soy | | p-value |
| --- | --- | --- | --- | --- | --- | --- | --- | --- |
|  |  | N | Mean (SEM) | N | Mean (SEM) | N | Mean (SEM) |  |
| TOTAL STANDARD SCORE | 3 months | 163 | 98.5 (0.7) | 147 | 96.7 (0.7) | 149 | 98.1 (0.7) | 0.154 |
|  | 12 months | 152 | 97.7 (0.6) | 135 | 96.8 (0.6) | 135 | 98.4 (0.6) | 0.152 |
|  | 24 months | 141 | 97.7 (1.0)_a_ | 116 | 94.0 (1.1)_ab_ | 116 | 95.0 (1.1)_b_ | **0.038** |
|  | 36 months | 128 | 104.8 (1.2)_a_ | 106 | 97.9 (1.3)_b_ | 97 | 97.9 (1.3)_b_ | **<0.001** |
|  | 48 months | 127 | 106.4 (1.2)_a_ | 105 | 101.6 (1.3)_b_ | 98 | 99.2 (1.3)_b_ | **<0.001** |
|  | 60 months | 130 | 105.6 (1.1)_a_ | 105 | 101.8 (1.2)_b_ | 99 | 99.9 (1.2)_b_ | **0.002** |
| AUDITORY COMPREHENSION | 3 months | 163 | 95.6 (0.6) | 148 | 94.4 (0.6) | 149 | 94.3 (0.7) | 0.261 |
|  | 12 months | 152 | 96.0 (0.6) | 135 | 95.1 (0.7) | 135 | 97.1 (0.7) | 0.105 |
|  | 24 months | 143 | 97.7 (1.1)_a_ | 117 | 92.6 (1.1)_b_ | 118 | 93.9 (1.1)_b_ | **0.003** |
|  | 36 months | 131 | 106.8 (1.2)_a_ | 114 | 99.8 (1.2)_b_ | 101 | 100.3 (1.3)_b_ | **<0.001** |
|  | 48 months | 132 | 107.1 (1.2)_a_ | 111 | 101.6 (1.3)_b_ | 101 | 99.8 (1.3)_b_ | **<0.001** |
|  | 60 months | 132 | 105.8 (1.2)_a_ | 107 | 103.4 (1.3)_ab_ | 102 | 100.6 (1.3)_b_ | **0.014** |
| EXPRESSIVE  COMMUNICATION | 3 months | 163 | 101.7 (0.9) | 147 | 99.3 (0.9) | 149 | 102.1 (1.0) | 0.088 |
|  | 12 months | 152 | 99.5 (0.6) | 136 | 99.0 (0.6) | 135 | 99.8 (0.6) | 0.636 |
|  | 24 months | 142 | 97.8 (1.0) | 118 | 96.4 (1.0) | 117 | 97.1 (1.1) | 0.617 |
|  | 36 months | 131 | 102.0 (1.2)_a_ | 107 | 96.0 (1.2)_b_ | 98 | 96.2 (1.3)_b_ | **<0.001** |
|  | 48 months | 128 | 104.4 (1.2)_a_ | 106 | 101.0 (1.3)_b_ | 99 | 98.9 (1.3)_b_ | **0.008** |
|  | 60 months | 131 | 104.3 (1.1)_a_ | 107 | 99.5 (1.2)_b_ | 100 | 99.4 (1.2)_b_ | **0.003** |

Least squares means (SEM) with different subscript letters within a row differ significantly at the p<0.05 level. P-values were Sidak-adjusted to control for model-wise type I error rates.
