# Supplementary material for: Developmental assessments during the first 5 years of life in infants fed breast milk, cow's milk formula, or soy formula
Source: Food Sci Nutr. 2020 May 13;8(7):3469–78. doi: 10.1002/fsn3.1630 (PMC7382202; doi:10.1002/fsn3.1630)
Supplement: Supplementary file 2 — Table, Supplemental Digital Content 1 [file FSN3-8-3469-s002.pdf]

Table, Supplemental Digital Content 1: Frequencies and Percentages of Developmental and Mental Health Disorders

	Breast	Milk	Soy	p-value
ADHD	1 (0.6)	4 (2.4)	3 (1.9)	0.369
Autism	0 (0.0)	1 (0.6)	2 (1.2)	0.213
Behavioral or Emotional Disorder	1 (0.6)	2 (1.2)	1 (0.6)	0.846
Developmental Delay	1 (0.6)	2 (1.2)	2 (1.2)	0.747
Sensory Disorder	1 (0.6)	0 (0.0)	2 (1.2)	0.318
Speech or Language Disorder	2 (1.2)	3 (1.8)	3 (1.9)	0.824
